# Molecular Cochaperones: Tumor Growth and Cancer Treatment

**DOI:** 10.1155/2013/217513

**Published:** 2013-04-17

**Authors:** Stuart K. Calderwood

**Affiliations:** Division of Molecular and Cellular Biology, Department of Radiation Oncology, Beth Israel Deaconess Medical Center, Harvard Medical School, 99 Brookline Avenue, Boston, MA 02215, USA

## Abstract

Molecular chaperones play important roles in all cellular organisms by maintaining the proteome in an optimally folded state. They appear to be at a premium in cancer cells whose evolution along the malignant pathways requires the fostering of cohorts of mutant proteins that are employed to overcome tumor suppressive regulation. To function at significant rates in cells, HSPs interact with cochaperones, proteins that assist in catalyzing individual steps in molecular chaperoning as well as in posttranslational modification and intracellular localization. We review current knowledge regarding the roles of chaperones such as heat shock protein 90 (Hsp90) and Hsp70 and their cochaperones in cancer. Cochaperones are potential targets for cancer therapy in themselves and can be used to assess the likely prognosis of individual malignancies. Hsp70 cochaperones Bag1, Bag3, and Hop play significant roles in the etiology of some cancers as do Hsp90 cochaperones Aha1, p23, Cdc37, and FKBP1. Others such as the J domain protein family, HspBP1, TTC4, and FKBPL appear to be associated with more benign tumor phenotypes. The key importance of cochaperones for many pathways of protein folding in cancer suggests high promise for the future development of novel pharmaceutical agents.

## 1. Introduction

*Chaperones/HSPs. *Molecular chaperones are a diverse group of proteins involved in the maintenance of other “client” proteins in folded and active conformations in all cellular organisms [1–5]. The term molecular chaperone is however generally reserved for proteins with a dedicated role in protein folding and refolding derived from the *HSPA* (HSP70), *HSPB *(small HSP), *HSPD* (Hsp60), *HSPC* (Hsp90), and *HSPH* (large HSP) gene families originally discovered as heat shock protein (HSP) genes [2, 6]. Products of these genes direct the folding of much of the proteome, resulting in the formation of proteins or protein complexes capable of metabolic functions in the cell. A subset of these proteins is also expressed at high levels in cells after proteotoxic stresses such as exposure to heat shock, heavy metals, alcohols and sodium arsenite [7–10]. Hence, they came to be known as heat shock proteins [7, 10]. Proteotoxic stresses lead to abundant levels of unfolded, aggregated, and ubiquibinated proteins, and cells respond to such an insult by abundant synthesis of HSPs capable of resolving these perturbations to the proteome [11]. These proteins are known to increase cell survival after stress both through direct chaperoning of malfolded proteins as well as inhibition of programmed cell death [12–14]. Altered demand for molecular chaperones is also associated with human diseases. For instance, in age-related degenerative diseases, aggregation-prone proteins accumulate and appear to exhaust the capacity of the molecular chaperone system [15–17]. Of more relevance for the current review, accumulation of mutated and overexpressed oncoproteins in cancer also leads to a demand for molecular chaperones and elevated levels of HSPs is characterize many malignancies [18–20]. This dependence on molecular chaperones appears to be a soft spot in the armor of the cancer cells and has led to the development of drugs aimed at depleting molecular chaperones and degrading the cancer proteome, leading to loss of viability of the tumors [21, 22]. In addition to their role in chaperoning the cancer cell proteome, HSPs are essential for evasion of a number of pathways of cell inactivation. For instance Hsp70 is involved in the inhibition of both caspase dependent apoptosis and senescence, key pathways in tumor suppression [14, 23–25]. The mechanisms which underlie the elevated expression of molecular chaperones of the *HSPA* and *HSPD* families in cancer are currently unclear but may involve the dysregulation of transcription factor HSF1 first shown to couple stress to HSP transcription [8, 26]. HSF1 is elevated and becomes activated in a wide range of cancers, its expression is coupled to the severity of disease, and it has been shown to be coupled to upstream signaling pathways in the malignant cell [27–31].

*Cochaperones. *As with many cellular proteins with dynamic function, HSP molecular chaperones do not appear to work alone and require the assistance of accessory proteins, known in this case as cochaperones [1, 3, 32–35] (Table 1). Three particular classes of chaperone appear of high significance in cancer and these include the *HSPA*, *HSPB,* and *HSPC* families [19]. We will discuss here the *HSPA* and *HSPC* molecular chaperone families and the associated co-chaperones. Each HSP class appears to be regulated by an individual cohort of co-chaperones and these molecules that may be significant factors in cancer and carcinogenesis and potential targets for therapy.

**Figure 1:**
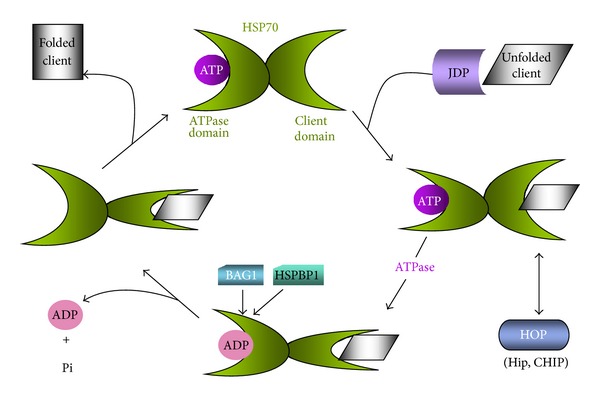
Reaction cycle for Hsp70 family polypeptides. Hsp70 proteins are portrayed as consisting of two major functional domains, including an N-terminal ATPase domain and a C-terminal client protein binding domain. When ATP occupies the ATPase domain, the C-terminal client protein-binding domain has weak affinity for clients. In the first step in chaperoning, the client protein (here depicted as unfolded) associated with a J domain protein (JDP) is able to bind Hsp70. This interaction causes allosteric changes in the N-terminal ATPase domain, ATP hydrolysis, and tight binding of substrate. Release of the client is then associated with nucleotide exchange loss of ADP and phosphate and replacement with ATP. In order to occur at a significant rate in cells nucleotide exchange factors such as Bag1 and HspBP1 are required. Released client is depicted as “folded.” However, the precise nature of the processes involved in achieving this state are not clear. We also show an alternative fate for Hsp70 client complexes involving the scaffold protein Hop. Hop can bind the extreme C-terminus of Hsp70 and couple it to other proteins such as Hsp90. Clients can thus be passed from Hsp70 to Hsp90 in a coordinated folding process.

Much has been discovered regarding the molecular and biochemical properties of *HSPA* family members such as Hsp70 and Hsc70. Hsp70 family members are described as being regulated by a bidirectional heterotrophic allosteric mechanism by a tautology between target polypeptides and adenosine nucleotides [3, 36]. Thus Hsp70, family proteins contain two major functional domains including an N-terminal nucleotide binding region and a C-terminal region that can interact with the hydrophobic residues in partially unfolded proteins (client binding domain) [5, 36–39] (Figure 1). “Empty” Hsp70 contains ATP in its N-terminal domain, and in this form the C-terminal domain can interact with suitably unfolded clients [3]. Binding is stabilized when the uptake of clients in the C-terminal domain triggers the ATPase activity of the N domain [3]. Clients are subsequently released, usually when refolded. This mechanism has been proposed to involve at least two modes of action, including (1) through stable “holding” of the client, Hsp70 maintains the levels of free unfolded client low enough to prevent aggregation; (2) inducing local unfolding in client protein domains and thus overcoming kinetic barriers to the native folded state. In the cell these biochemical properties of Hsp70 and Co. is regulated by co-chaperones that can couple activity to cell physiology/pathology [32, 40]. 

For Hsp70, co-chaperones include a large family of J domain proteins that can bind to specific substrates and foster association of such clients with Hsp70 [40, 41]. These proteins contain a J domain capable of interacting with the ATP domain of the Hsp70s and a client binding domain that can associate with unfolded proteins and transfer them to the client binding site of Hsp70, resulting in a multifold stimulation in the ATPase activity of client-bound Hsp70 [41–43]. There are at least 49 members of the human J domain protein family [44–46]. Following the “client holding” stage of the cycle, the next step is client release and perhaps refolding. In order for Hsp70 to release clients, ADP must dissociate from the N-terminal domain, a relatively gradual reaction that can however be strongly stimulated by nucleotide exchange factors such as BAG domain proteins [45–48]. The BAG (Bcl2-associated athanogene) domain interacts with the ATPase domain of Hsp70 and stimulates ADP release permitting client efflux by allosteric regulation of the N-terminal domain [47, 48]. Other nucleotide exchange factors include HspBP1 [49, 50]. The cytoplasmic members of the hspa family also contain a TPR domain binding (TDB) site at the extreme C-terminus. The TPR (tetratricopeptide) domain is a protein interaction motif found in a range of proteins many of which interact with molecular chaperones Hsp70 and Hsp90 [51]. In the case of Hsp70, such TPR domain proteins include the scaffold protein Hop/Sti (Hop: Hsp70/Hsp90 organizing protein) and the ubiquitin E3 ligase CHIP [52–54]. 

HSPC (Hsp90) proteins, although bearing minimal sequence conservation with the HSPA family, have some biochemical properties in common with the Hsp70 proteins [55–57]. The HSPC family includes four major members, including two cytoplasmic proteins Hsp90*α* and Hsp90*β*, an ER resident member glucose-regulated protein 94 (grp94) and a mitochondrially located member TRAP1 [57–60]. Hsp90*α* and Hsp90*β* are of most significance in cancer where they are expressed to very high levels and are significant targets for drug development [18, 21, 61, 62]. These molecules have in common with Hsp70 the ability to bind and hydrolyze ATP and to bind and modify the conformations of clients [63]. Hsp90 proteins function as dimers and the ATP binding, and hydrolysis cycles regulate dimerization and client binding as with Hsp70 [56, 64–67]. The nature of the client interaction domain is not entirely clear although it is thought to bind the amino acid and middle domains on the outside of the dimer [68, 69]. In addition, cytoplasmic Hsp90 proteins also contain a TPR domain binding site at the C-terminus. Hsp90 interacts with an array of co-chaperones. These include p23/Sba1, a protein with intrinsic chaperone activity that stabilizes the closed conformation of Hsp90 by inhibiting ATPase activity and thus prolongs interaction with clients such as steroid hormones [70, 71]. Another key co-chaperone is p50/*Cell Division Cycle 37 *(Cdc37) that binds the N-terminal ATP binding domain, inhibits ATPase activity, and is of particular significance in interaction with protein kinases [72–75]. While p23 and Cdc37 appear to function by stabilizing Hsp90 client interactions, another co-chaperone Sgt1 appears to function at the beginning of the cycle binding ATP-free Hsp90 and helping to recruit clients to the chaperone in an analogous way to the function of JDPs in Hsp70 [76]. The other core co-chaperone is *Activator of the Hsp90 ATPase* (Aha1), a protein that binds Hsp90 in the middle domain and triggers ATPase activity and perhaps dissociation of clients [77, 78]. In addition to this core group, a large array of TPR domain co-chaperones bind to the C-terminus TDB motif of Hsp90 and can modulate chaperoning functions in specific clients, including scaffold protein Hop, protein phosphatase PP5, immunophilins FKBP1, FKBP2, Cyp4, and TPR domain proteins TTC4, TTC5, and XAP2/AIPL1 [68]. 

The TBD motifs of Hsp70 and Hsp90 proteins are key to determining the role of both proteins in intracellular protein quality control as well as more subtle interaction with bound clients. When Hsp70 binds to the multiple TPR domain scaffold protein Hop on its specific recognition site, Hsp90 can also bind to another TPR motif in Hop [79, 80]. Such coupling permits a coordinated pathway of remodeling of some clients, with initial folding of nascent proteins by Hsp70/HDJ complexes commencing on the ribosome, preceding fine tuning of client conformation by Hsp90/p23/Cdc37/immunophilin complexes and full maturation of functional polypeptides [73]. Alternatively, protein quality control can be biased towards proteolysis and disposal of damaged proteins when the E3 ligase CHIP binds the TBD motifs of either Hsp70 or Hsp90 [54, 81, 82]. Triage between the various arms of protein quality control involves complex regulatory decision making. For instance, formation of the Hsp70/Hop/Hsp90 complex is favored by the nucleotide exchange factor Bag1 [45]. Alternatively, Bag3 favors the disposal of polyubiquitinated proteins by macroautophagy [83, 84]. As mentioned above, Hsp90 can bind a range of co-chaperones through the C-terminal TBD motif and can affect changes in phosphorylation of clients by PP5 and maturation of clients such as steroid hormones by immunophilins Cyp40, FKBP1, and FKBP2 [68]. Although the immunophilins are able to effect proline isomerization as a basic function, these properties do not seem essential in Hsp90 cochaperone mode [85, 86]. Other mechanisms seem to be involved. TTC 5 is an interesting molecule with potentially profound roles in cell physiology. This protein contains 6 TPR motifs and binds to transcriptional co-activators, activating transcription by factors such as p53 and HSF1 [87, 88]. Binding to the TBD motifs on Hsp70 or Hsp90 might thus permit coupling of protein unfolding in stress or quality control to transcriptional remodeling.

## 2. Hsp70 Cochaperones and Cancer

### 2.1. BAG Domain Proteins

As mentioned above, BAG domain proteins (Bag1-6) can associate with the nucleotide binding domain of Hsp70 family members, stimulate nucleotide exchange, and thus promote molecular chaperone activity (Figure 1) [3]. Bag1-6 are multidomain proteins, each of which contains the Hsp70-binding BAG domain close to the C-terminus [46, 47, 89, 90]. The two family members with most significance in cancer appear to be Bag1 and Bag3. Bag1 also contains a UBL domain that can bind polyubiquitinated proteins and indeed has been shown to bias protein quality control towards proteolytic degradation through its ability to bind polyubiquitinated proteins [3]. Bag3 contains WW and proline rich repeat sequences that appear to permit it to interact with cell signaling molecules [46]. Indeed, Bag3 can interact with the molecular chaperone HspB8 and mediate macroautophagy [83]. Another difference between Bag1 and Bag3 is that while Bag1 is constitutively expressed, Bag3 is conditionally inducible and indeed responds to the stress-inducible transcription factor HSF1 [91]. Elevated expression of Bag1 and Bag3 in each case signals a poor prognosis for cancer bearing patients. This effect may be related to the ability of BAG/Hsp70 complexes to inhibit apoptosis [90–92]. Indeed double knockdown of Bag1 and Bag3 in acute myeloid leukemia caused loss of antiapoptotic proteins Bcl2, Bcl-XL, Mcl1, and phosphor-ERK1/2 [92]. The roles of Bag1 and Bag3 are somewhat complicated by their opposing role in protein quality control, with Bag1/Hsp70 favoring proteasomal degradation of clients (such as BCR-ABL oncoproteins) and Bag3 competing for binding to Hsp70 and deterring entry into the proteasome pathway [45, 46]. For instance, complexing of Bag3 with Hsp70 can protect oncogenic IKK gamma from proteasomal degradation, increase flux through the NF kappa B pathway, and increase cell growth and survival [93]. 

### 2.2. HspBP1

Similar to the BAG domain proteins, HspBP1 is also a nucleotide exchange factor that can stimulate the ATPase cycle of Hsp70 (Figure 1). Although both proteins induce nucleotide exchange, the mechanisms employed for this activity appear to involve contrasting interactions with the ATPase domain [94]. In addition, HspBP1 appears to be able to oppose the proteolytic pathway of protein quality control and binding of the protein to the ATPase domain of Hsp70 inhibits the ubiquitin ligase activity of CHIP when attached to the TBP domain of Hsp70 [95]. In contrast to the BAG proteins, HspBP1 appears to play a suppressive role in a number of types of cancer [96]. HspBP1 levels are elevated in breast tissue and inversely related to aggressiveness [96]. In addition some chemotherapeutic agents can increase cytotoxicity by inhibiting Hsp70 function [97]. Hsp70 appears to inhibit a unique pathway of cell death in tumor cells involving lysosomal membrane permeabilization and activation of caspase 3 [98, 99]. HspBP1 appears to oppose this Hsp70-regulated pathway [97]. 

### 2.3. J Domain Proteins

JDPs are the most abundant family of Hsp70 co-chaperones with at least 49 members [44]. The founder member of the family is the *E. coli* DNAJ, which along with the prokaryotic Hsp70 (DNAK) and nucleotide exchange factor (Grpe) is responsible for the bacterial Hsp70 cycle as in Figure 1 [55, 100]. JDPs characteristically function by locating client proteins and ensuring their tight binding to Hsp70 [3, 44]. This is assisted by the ability of JDPs to stimulate the ATPase activity of the Hsp70 chaperone and lock the client into the closed peptide binding domain [44]. All JDPs contain the 70 amino acid J domain, which is essential for interaction of the protein with Hsp70. There are three families of JDP, including types I, II, and III [44]. JDPs tend to be either elevated to distinctly high levels or deregulated in cancer [101]. However, the majority of JDP, at least in studies carried out so far, appear to be tumor suppressive in nature [44]. Notable examples of tumor suppressive JDP include TID1/DNAJA3 that functions in the mitochondrial matrix as an inhibitor of carcinogenesis and *mammalian relative of DnaJ* (MRJ) that negatively regulates breast cancer malignancy and reduces b-catenin signaling [102–105].

### 2.4. Hop

Hop, also known as STIP1, mediates Hsp70/Hsp90 interactions through their TBD domains [106] (Figure 1). Only a limited amount of information is available regarding the possible involvement of Hop in cancer. However, increased levels of Hop were observed in human colon cancer, associated with increased Hsp70, Hsp90, and Hop/Hsp70/Hsp90 complexes [107]. Hop expression is also increased in hepatocellular carcinoma [108]. Interestingly, knockdown of Hop by RNA targeting in pancreatic cancer cells reduced the levels of proteins that have been previously isolated as Hsp90 clients in cancer such as HER2, Bcr-Abl, c-Met, and v-Src, suggesting that Hop ablation in cancer cells could be functionally similar to Hsp90 inhibition which decreases tumor growth by reducing levels of key oncoproteins [18, 22, 109]. As mentioned above, Hop is a poly-TPR domain scaffold protein that couples Hsp90 to the Hsp70 folding cycle as well as to other co-chaperones, and loss of Hop may be functionally similar to deletion of the TBP domain of Hsp90, a modification that ablates chaperone function [106]. In addition, Hop knockdown also led to the loss of matrix metalloproteinase 2 (MMP-2) and a decrease in cancer cell migration, also consonant with a permissive role for Hop in cancer progression [109]. Hop is thus a co-chaperone for both Hsp70 and Hsp90, a coordinator of Hsp70/Hsp90 interaction, a scaffold for binding of other co-chaperones, and a potential target in cancer. 

## 3. Hsp90 Cochaperones and Cancer

### 3.1. P23

**Figure 2:**
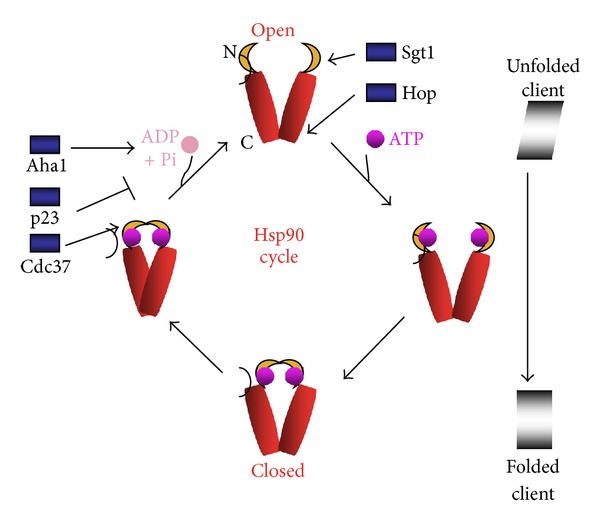
Reaction Cycle for Hsp90. Hsp90 functions as a dimer in a cycle of client binding, ATP binding, ATPase activity, and nucleotide exchange as with Hsp70. The dimers are in an open conformation when empty and a more closed conformation when ATP binds. Substrate binding is assisted by co-chaperone Sgt1. For Hsp90, in which prolonged “holding” of clients seems an important component of its cellular function, the co-chaperones p23 and Cdc37 inhibit ATPase activity and stabilize Hsp90 client complexes. For nucleotide exchange to take place after ATPase activity eventually occurs, Aha1 functions as an exchange factor. As with Hsp70, triage of the protein between pathways of protein quality control involves scaffold protein Hop that binds the C-terminal TPR domain-binding motif (TBD). Through the TBP, Hsp90 can interact with a range of TPR domain containing co-chaperones that regulate intracellular function.

P23 is an inhibitor of the ATPase activity of Hsp90 (Figure 2) and through this function can promote sustained interaction between hsp90 and a wide range of clients such as steroid hormone receptors and HSF1 [70, 110]. Recent studies point to a role for p23 in tumor progression and cancer formation. High levels of p23 were associated with increased metastasis in breast cancer and indicated a poor prognosis including enhanced disease recurrence [111]. In addition, p23 targets genes involved in drug resistance and metastasis such as PMP22, ABCC3, AGR2, Sox2, TM4SF1, and NUPR were expressed to high levels in p23 overexpressing mammary carcinoma cells [111]. p23 could also be involved in the incidence of prostate cancer as a key component of the androgen receptor activity [112]. Interestingly, these effects may involve both Hsp90-dependent and Hsp90-independent effects. Hsp90-independent roles for p23 have been shown previously [113]. Another mechanism for increasing cancer incidence may involve inhibition of apoptosis in malignant cells. p23 is overexpressed in acute lymphoblastic leukemia (ALL) and functions as an inhibitor of chemotherapy induced apoptosis [114]. These effects may be connected to loss of the microRNA species has-miR-101 in childhood ALL cases and concomitant p23 dysregulation [114]. Interestingly, a novel plant product known as gedunin has been isolated that can bind p23, block its chaperoning and transcriptional activities, and lead to programmed cell death in malignant cells [115]. 

### 3.2. Sgt1

Sgt1 is a CHORD domain protein that acts early in the ATPase cycle of Hsp90 and may help to recruit client proteins in a similar manner to JDP for Hsp70 [116]. In addition, Sgt1 can bind to Hsp70 leading to the formation of an Sgt1/Hsp90/Hsp70 chaperone complex important in the function of leucine rich repeat proteins [117]. However, although Sgt1 shares sequence similarities with p23, few reports of a role for the protein in cancer are currently available. Sgt1 seems to be an important HSP co-chaperone in innate immune signaling through the intracellular NLR pathway and in kinetochore function [118, 119].

### 3.3. P50/Cdc37

Cdc37 appears to play a highly significant role in cancer and its forced expression in transgenic mice leads to prostatic hyperplasia and, when expressed in conjunction with the oncogene c-Myc, to prostate cancer [120, 121]. Cdc37 is also expressed to high level in other types of malignancy such as anaplastic large cell lymphoma, acute myelocytic leukemia, hepatocellular carcinoma, and multiple myeloma [122–125]. The key biochemical function of Cdc37 appears to slow down the ATPase cycle of the Hsp90 complex and extend the holding time of the client [73]. Cdc37 and Hsp90 form triple complexes with many proteins, being associated with protein kinases in particular [126, 127]. Such protein kinases include a long list of enzymes involved in promoting cell growth, including receptor tyrosine kinases epidermal growth factor (EGFR) and MET, nonreceptor tyrosine kinases SRC and LCK, and serine/threonine kinases RAF1, AKT1, IKK CDC2, and CDK2 (see [73, 74]). Not surprisingly, reduction in Cdc37 levels by RNA interference had a profound effect in reducing tumor cell growth [128, 129]. In prostate carcinoma, Cdc37 knockdown inactivated cell growth and sensitized tumors to Hsp90 inhibitors [128]. It is not clear why Cdc37 is selectively carcinogenic in prostate as opposed to other tissues. One possibility is that the androgen receptor, one of the few nonkinase clients of Cdc37, is activated by elevated levels of Cdc37 [130]. However, Cdc37 knockdown inhibited growth of androgen receptor negative Prostate Carcinoma (PC-3 and DU-145) as effectively as it affected androgen-requiring LnCap cells [128]. In these cells, Cdc37 effectively inhibited both the ERK and Akt pathways as well as EGFR signaling [128] (J. Cheng & S.K. Calderwood, in preparation). Cdc37 therefore seems to be a strong molecular candidate for targeted therapy, particularly in prostate carcinoma [73].

### 3.4. Aha1

While many chaperones function to slow down the ATPase cycle of Hsp90, Aha1 triggers ATPase activity and release of client proteins from the complex [77]. Despite this, Aha1 appears to be involved in increasing the activity of c-Src by Hsp90 chaperone machines and maintaining signaling activity [131]. Aha1 mRNA levels are elevated in lymphoblast and testicular germ cells, although few studies have addressed levels in cancer [132]. However, Aha1 concentrations appear high in promyelocytic leukemia and Daudi Burkett's lymphoma cells [132]. Holmes et al. studied Aha1 protein levels in a range of human cancer cells and found markedly different levels in the different cell lines [133]. Treatment with the Hsp90 inhibitory drug 17-AAG increased Ah1 levels, an effect that appeared to reflect the activation of HSF1 by the Hsp90 ablation [133]. As might be predicted, reduction in Aha1 levels led to sensitization of cells to 17-AAG [133]. 

## 4. Hsp90 Interaction with TPR Domain Proteins

A number of proteins have been shown to bind to the TBD domains of Hsp90, including the scaffold protein Hop as mentioned above. These interactions have been intensively studied in regard to the regulation of steroid hormone receptors [63]. Hsp90 and co-chaperones are thought to be continuously required to maintain proteins such as glucocorticoid receptor (GR), mineralocorticoid receptor, and progesterone receptor in stable conformations receptive to activation by their respective ligands [32, 63, 134]. Receptors such as estrogen receptor and androgen receptor appear less prone to regulation by chaperone complexes [134]. TPR domain proteins that interact with the C-terminal TBD of Hsp90 include protein phosphatase 5 (PP5), immunophilins FKBP1/2 and Cyp40, and TPR additional domain proteins such as TTC4, TTC5, TPR2, XAP2, and AIPL1 [134].

*Protein Phosphatase 5 (PP5).* PP5 is a serine/threonine phosphatase and is a member of the PPP phosphatase family that also includes PP1 and PPA2 [139–141]. Clearly, association with a protein phosphatase has considerable potential for modulating the properties of Hsp90 complexes. Indeed, binding of PP5 to Hsp90 has potentially pleiotropic effects as the enzyme may modify the phosphorylation of Hsp90 itself as well as modulating phosphorylated sites on other co-chaperones and on its bound clients [142, 143]. PP5 can dephosphorylate Hsp90 and thus positively regulate its molecular chaperone activities [142, 144]. In addition, Hsp90-bound PP5 dephosphorylates the key co-chaperone Cdc37 on the residue serine 13, essential for intracellular function and thus reduces its ability to chaperone some of its many kinase clients [145]. The metabolic activities of chaperoned clients may be positively or negatively regulated by Hsp90-associated PP5, depending on the nature of the phosphorylation sites involved. For instance, in lipogenesis, PP5 can simultaneously activate GR and repress Peroxisome Proliferator Activated Receptor-*γ* (PPAR-*γ*) through dephosphorylation of certain serine residues [140]. In addition, Hsp90-PP5 interactions also influence cell cycle progression and DNA repair pathways through dephosphorylation of key Ser/Thr residues in DNA-dependent protein kinase and ATR [146, 147]. Thus, PP5 may play a key role in regulating many aspects of chaperone complexes in cancer although it is currently not clear what the overall effect of Hsp90-associated PP5 might be. 

*Immunophilins.* Immunophilins are proteins that bind to immunosuppressive drugs such as cyclosporine and FK506 and have common domains with peptidyl-prolyl cis-trans isomerase (PPIase) activity [162]. At least three immunophilins with TPR domains, FKBP1, FKBP2, and Cyp40, are known to bind the TBD of Hsp90 and act as co-chaperones [68]. Cyp40 is present in complexes between Hsp90 and a number of steroid hormones such as GR and PR and increased transcriptional activity of the hormones when overexpressed [134, 163]. Activity appears to require the TPR and PPIase domains although a role for PPIase activity in the co-chaperone properties of Cyp40 has not been demonstrated [85, 86]. Cyp40 and other immunophilins may play a role in hormone-dependent malignancies such as prostate and breast cancer. Cyp40 and FKBP1 are elevated in prostate cancer compared to normal cells, positively regulate androgen dependent prostate cancer growth, and increase AR-dependent transcription [164, 165]. Growth of such cancers is suppressed by cyclosporine A and FK506, the immunophilin ligands that inhibited several stages of AR signaling [164, 165]. It had been shown previously that Cyp40 and FKBP2 are increased in mammary carcinoma cells by estradiol and that the antiestrogen drug ICI 182, 780 antagonized these increases [166]. 

FK506 binding proteins (FKBPs) including FKBP1 and FKBP2 are related proteins that, like Cyp40, contain a PPIase domain closely apposed to a TPR domain [135, 136]. As with Cyp40, co-chaperone activity is associated with the PPIase domain but does not require PPIase activity [137]. For FKBP2 at least, sequences in the PPIase domain may interact with the ligand binding domain of Hsp90 [137]. FKBP2 may function by increasing nuclear transport through interaction with the motor protein dynein. Although structurally similar, these two Hsp90 co-chaperones appear to have opposing effects on nuclear receptor transcriptional activity, with the tightly binding FKBP1 inhibiting activity and the more loosely associated FKBP2 activating at least in the case of MR, GR, PR and AR [134, 137]. These immunophilins may compete for binding to nuclear receptor-chaperone complexes through differential binding to the TBD of Hsp90, and for instances FKBP2 can reverse the inhibitory effects of FKBP1 on GR activity [167]. However, Cyp40 was unable to oppose the trans-inhibitory effects of FKBP1 [134]. Recently another member of the FKBP family has emerged FKBP-like or FKBPL that may have high significance in cancer [138, 168, 169]. FKBPL has a conserved TPR domain although the PPIase domain is divergent compared with FKPP1, 2 [138]. 

As mentioned above-immunophilins appear to be significant in hormone-dependent cancer, and FKBP1 in particular is enriched in prostate cancer as opposed to benign tumors and stimulates androgen-dependent transcription and growth [164–166, 170, 171]. FKBP2 appears to play a role in breast cancer and treatment with estradiol led to a 14-fold increase in expression [166]. FKBPL is associated with ER in breast cancer, and increased levels of the protein indicate a good prognosis in the case of this disease [138]. 

*TTC4.* Tetraticopeptide 4 (TTC4) is another TPR domain protein and was originally discovered in a screen for loss of heterozygosity in the chromosomal 1p31 region associated with breast cancer and was thus implicated as a tumor suppressor gene [148, 149]. TTC4 appears to function in the nucleus and has been implicated as a component of a complex containing the histone acetyltransferase MYST/MOF and in the assembly of transcriptional initiation factor TFIIIB [149–152]. TTC4 is also a potential Hsp90 binding protein and appears to have an important role in linking Hsp90 function to replication [153], TTC4 may thus have a role in multiple nuclear functions including transcription and replication and these may be linked to its tumor suppressor properties.

*TTC5.* Tetraticopeptide 5 (TTC5) also known as stress-responsive activator of p300 (Strap) contains six TPR domains, and with these multiple interaction domains could thus play a role as a scaffold protein in a similar way to Hop [87, 88]. TTC5/Strap binds to the histone acetylase p300 and is implicated in the activation of transcription in response to stresses such as DNA damage and heat shock [88, 154, 155]. p300 is a cofactor for a wide range of transcription factors, as well as forming complexes with an array of other proteins and can modify both the associated factors as well as histone H4 by acetylation [156]. Indeed, TTC5/Strap is implicated in the regulation of GR as with other co-chaperones such as Cyp40, FKBP1, and FKBP2 and could potentially couple molecular chaperones and stress to transcriptional activation [157]. TTC5/Strap may thus play a role in transcriptional regulation during stress responses to heat shock or DNA damage although currently no evidence appears to link this co-chaperone to cancer. 

*XAP2/AIP.* Another TPR domain containing Hsp90 binding co-chaperone is XAP2, also known as AIP [158]. This protein is a member of the immunophilin family and regulates activity of steroid hormone receptors such as the aryl hydrocarbon receptor and the estrogen receptor *α* (ER*α*) [159, 160]. The co-chaperone could potentially play a role in breast cancer through its negative regulation of ER*α* [159]. ER can stimulate mammary cancer growth but can also function as an inhibitor of metastasis, and its exact role in cancer would thus be difficult to predict [161]. 

## 5. Overview: Cochaperones and Cancer

**Figure 3:**
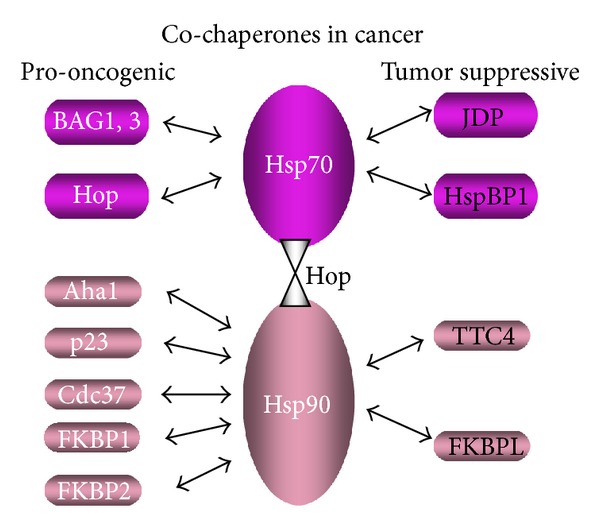
Oncogenic or tumor suppressive influence of molecular co-chaperones. (Hop is shown twice in the cartoon to depict its role in bridging Hsp70 and Hsp90 as well as its pro-oncogenic influence).

As co-chaperones are essential for significant molecular chaperone activity *in vitro*, one might predict a uniform procancer role for these molecules as with the primary chaperones. However, their role in cancer appears to be complex. It is thus evident that a number of co-chaperones are overexpressed in cancers and signal a poor prognosis in patients and may be candidates for the development of novel approaches to cancer therapy. Hop, p23, p50/Cdc37, Aha1, FKBP1, and FKBP2 appear to be intimately involved in the chaperoning of molecules involved in cancer incidence and progression (Figure 3). Cancers appear to become addicted to these co-chaperones in a similar way to their dependence on the primary chaperones, requiring these co-factors to maintain elevated levels of oncogenes which are often mutated during carcinogenesis [18, 21]. In addition, the BAG domain proteins appear to indicate a poor prognosis for cancer patients due to their *inhibition of apoptosis*—one of the key hallmarks of cancer [172, 173]. However, a sizable number of the co-chaperones, including HspBP1, the JDP family proteins, FKBPL, and TTC4 appear to signal a good prognosis in cancer suggesting that they may have tumor suppressive functions (Figure 3). As mentioned above, TTC4 in particular was identified in a loss of heterozygosity screen for tumor suppressor genes. 

## 6. Molecular Chaperones and Cochaperones in Cancer Treatment

The targeting of Hsp90 in cancer was initially stimulated by the availability of drugs such as geldanamycin and radicicol that bind to and inhibit its ATPase domain [174, 175]. Hsp90 has since become a heavily targeted molecule, and several generations of anticancer drugs including the 17-allylamino-17-demethoxygeldanomycin (17-AAG) showed promise in cancer treatment [176, 177]. More modern synthetic drugs have recently become available that have solved some of the early problems with drug toxicity, and progress in this area can be expected [174]. Hsp70/*HSPA* family members also have considerable promise as targets in cancer and proteins such as Hsp72, Hsp70.2, and mortalin are increased in breast cancer and when inhibited elicit apoptosis [178–180]. In addition, the Hsp70 family members appear essential in chaperoning oncogenic proteins as is observed with Hsp90, and compounds capable of inhibiting the Hsp70 chaperones are beginning to emerge [181]. In addition, a recent pharmacological study of AR signaling using gene expression profiling indicated two more classes of drugs that might inhibit chaperone/co-chaperone interactions. Two drug families, centering on the natural compounds celastrol and gedunin, were uncovered [182]. Celastrol was shown to disrupt the function of the Hsp90/Cdc37 complex, a key growth-requiring pathway in prostate cancer and thus is a promising agent for this disease [183]. The drug is however somewhat lacking in specificity and also directly inhibits I*κ*B kinase and the proteasome as well as activating HSF1 [184]. As mentioned above, gedunin inhibits p23, another prooncogenic Hsp90 co-chaperone [115]. Therefore, although in its infancy, the concept of targeting co-chaperones in cancer treatment seems feasible and practical. 

## Figures and Tables

**Table 1:** Cochaperones.

Name	Function	Conserved domain	Role in cancer	Associated proteins	Reference
Hsp70					

JDP	Substrate selection	J	Supp	—	[101–105]
HspBP1	Nucleotide exchange	—	Supp	—	[96–99]
Bag1	Nucleotide exchange	BAG, UBL	Onc		[92]
Bag3	Nucleotide exchange	BAG, WW	Onc	HspB8	[90, 91, 93]
Hop	Adaptor	TPR	Onc	TDB proteins	[107–109]

Hsp90					

Sgt1	Substrate selection	CHORD	—	Hsp70	[116–119]
P23	ATPase inhibitor	p23	Onc	—	[112–115]
Aha1	ATPase inhibitor	Aha1	Onc	—	[131, 132]
Cdc37	ATPase inhibitor	Hsp90 bd	Onc	Kinases	[120–129]
Hop	Adaptor	TPR	Onc	TDB	[107–109]
Cyp40	Immunophilin	TPR, PPiase	—	TDB	[32, 134]
FKBP1	Immunophilin	TPR, PPiase	Onc	TDB	[135, 136]
FKBP2	Immunophilin	TPR, PPiase	Onc	TDB, dynein	[134, 137, 138]
PP5	Protein phosphatase	TPR	—	TDB	[139–147]
TTC4	Adaptor, replication	TPR	Supp	TDB, Myst, MOF	[148–153]
TTC5	Adaptor, transcription	TPR	—	TDB, p300	[88, 154–157]
XAP2	Nuclear receptors	TPR	—	TDB	[158–161]
